# Using the Daydreaming Frequency Scale to Investigate the Relationships between Mind-Wandering, Psychological Well-Being, and Present-Moment Awareness

**DOI:** 10.3389/fpsyg.2012.00363

**Published:** 2012-09-25

**Authors:** David Stawarczyk, Steve Majerus, Martial Van der Linden, Arnaud D’Argembeau

**Affiliations:** ^1^Department of Psychology – Cognition and Behavior, University of LiègeLiège, Belgium; ^2^Fonds National de la Recherche ScientifiqueBrussels, Belgium; ^3^Cognitive Psychopathology and Neuropsychology Unit, University of GenevaGeneva, Switzerland

**Keywords:** mind-wandering, daydreaming, mindful awareness, encoding style, psychological distress, well-being

## Abstract

Recent findings have shown that mind-wandering – the occurrence of stimulus-independent and task-unrelated thoughts – is associated with negative affect and lower psychological well-being. However, it remains unclear whether this relationship is due to the occurrence of mind-wandering *per se* or to the fact that people who mind wander more tend to be generally less attentive to present-moment experience. In three studies, we first validate a French translation of a retrospective self-report questionnaire widely used to assess the general occurrence of mind-wandering in daily life – the Daydreaming Frequency Scale. Using this questionnaire, we then show that the relationship between mind-wandering frequency and psychological distress is fully accounted for by individual differences in dispositional mindful awareness and encoding style. These findings suggest that it may not be mind-wandering *per se* that is responsible for psychological distress, but rather the general tendency to be less aware and attentive to the present-moment. Thus, although mind-wandering and present-moment awareness are related constructs, they are not reducible to one another, and are distinguishable in terms of their relationship with psychological well-being.

## Introduction

When reading a book, driving to work, or performing other common daily tasks, our mind frequently drifts away from our current activity and focuses instead on internal thoughts and images that are unrelated to the present situation (e.g., remembrances of the past or thoughts about future events). This particular kind of thought, often referred to as mind-wandering or daydreaming, can be defined as stimulus-independent and task-unrelated thoughts (SITUTs), in the sense that their content (i) is not the direct reflection of current sensory input and (ii) is unrelated to the task being performed at the moment of their occurrence (Stawarczyk et al., 2011a,b). Experience sampling studies have shown that SITUTs are an ubiquitous phenomenon experienced by virtually everyone (Singer and McCraven, 1961), and cover 30–50% of our daily thinking time (Kane et al., 2007; Killingsworth and Gilbert, 2010).

This frequent occurrence of SITUTs in daily life has led to the suggestion that these thoughts serve a purpose in terms of ongoing cognitive processes (Smallwood and Schooler, 2006). Research focusing on the content of SITUTs has demonstrated that most of these thoughts are self-related (Baird et al., 2011; Smallwood et al., 2011), temporally oriented toward the future (Smallwood et al., 2009b), and directed toward planning and preparing for impending events (Baird et al., 2011; Stawarczyk et al., 2011a). It has therefore been proposed that SITUTs play an important role in the processing of personal goals and concerns (Smallwood and Schooler, 2006; Klinger, 2009). SITUTs may allow us to manipulate and organize internal information, to solve problems that require computation over long periods of time, and to create effective plans governing our future behaviors in concordance with our personal aims and aspirations (Binder et al., 1999).

While SITUTs may support specific cognitive processes, a growing body of research also indicates that the occurrence of SITUTs is not without deleterious consequences (for recent reviews, see Klinger, 2009; McVay and Kane, 2010; Christoff et al., 2011; Schooler et al., 2011; Smallwood et al., 2012). SITUTs have been associated with decreased performance on a wide array of activities, including reading (Smallwood, 2011; McVay and Kane, 2012b), car driving (He et al., 2011), paying attention during lectures(Lindquist and McLean, 2011; Risko et al., 2012), reaction time tasks (Smallwood et al., 2004a; McVay and Kane, 2012a), and memory tasks (Smallwood et al., 2003, 2004c). Furthermore, recent electrophysiological studies have shown that sensory evoked potentials to both task-related and task-unrelated stimuli were decreased while people were experiencing SITUTs in comparison to when their attention was fully focused on-task (Barron et al., 2011; Kam et al., 2011). Together, these findings suggest that SITUTs are a resource-consuming phenomenon in which attention to sensory information is reduced in favor of internally generated cognitions, resulting in a state of perceptual decoupling from the here and now (Smallwood and Schooler, 2006; Smallwood, 2010, 2011; Schooler et al., 2011).

Besides their influence on present-moment attention, SITUTs also seem to impact psychological well-being and mental health. There is substantial evidence that people who experience more SITUTs suffer from increased depressive symptomatology (Giambra and Traynor, 1978; Watts et al., 1988; Smallwood et al., 2004b, 2007; Burg and Michalak, 2011) and report less life satisfaction (Mar et al., 2012). Of particular interest, Killingsworth and Gilbert (2010) recently used an experience sampling procedure to assess the occurrence of SITUTs in the daily life of 2250 participants. They found that mind-wandering sampled at time *t*−1 was a predictor of a lower mood at time *t*, whereas mood at time *t* was unrelated to the presence of mind-wandering at time *t *+ 1. Although some laboratory findings have recently revealed that this relationship might not be totally unilateral (Smallwood et al., 2009a; Smallwood and O’Connor, 2011), the results by Killingsworth and Gilbert (2010) suggest that SITUTs might be one of the causes rather than the consequence of unhappiness and psychological distress. Other research suggests that factors such as the valence of thought content, repetitiveness, and level of construal (abstract versus specific) play important roles in determining the negative emotional consequences of SITUTs (Watkins, 2008, 2010).

In short, although mind-wandering seems to serve adaptive functions such as planning and preparing for future events, it also tends to be associated with negative affect and lower psychological well-being. It remains unclear, however, whether the relationship between mind-wandering and psychological well-being that has been documented in previous studies is due to the occurrence of SITUTs *per se*. Recent findings have revealed that the frequency of SITUTs is higher in individuals who are less aware of their present-moment experience, as assessed by dispositional measures of mindful awareness (Burg and Michalak, 2011; Mrazek et al., 2012), and it is well-known from research on trait mindfulness that decreased attention to the present-moment is associated with lower psychological well-being (Brown and Ryan, 2003; Brown et al., 2007; Jermann et al., 2009; Keng et al., 2011). Mindfulness involves the ability to anchor one’s attention on what is occurring (e.g., sensations, thoughts, feelings), and the ability to intentionally switch attention from one aspect of experience to another (Brown et al., 2007; Keng et al., 2011). People who are less mindful may thus be less aware of SITUTs when they occur and less able to regulate and manage these thoughts (e.g., to view them as passing mental events and to let them go), thus increasing their negative emotional impact (Frewen et al., 2008; Keng et al., 2011)^1^. Therefore, it could be that the relationship between SITUT frequency and psychological distress is not due to the occurrence of mind-wandering *per se*, but instead to the fact that people who present more mind-wandering episodes are generally less attentive to present-moment experience (and thus less able to regulate their thoughts).

In the present study, we tested this hypothesis by examining the relationships between various self-report instruments of general daily life experiences that included measures of psychological distress and SITUT frequency, as well as two measures reflective of a general tendency to have one’s attention decoupled from the present-moment: (i) dispositional mindful awareness, which indicates the degree to which individuals are attending to the here and now (Brown and Ryan, 2003; Jermann et al., 2009), and (ii) encoding style, which indicates the degree to which individuals pay careful attention to the external environment (i.e., external encoding style) versus less careful attention, caused by attending relatively more to internal mental processes (i.e., internal encoding style; Lewicki, 2005; Herndon, 2008; Billieux et al., 2009). We predicted that if a general tendency for decreased attention to the present-moment is responsible for the relationship between SITUTs and psychological distress, then SITUT frequency should not remain a significant predictor of psychological well-being once the influence of mindful awareness and encoding style had been taken into account. To test this hypothesis, we performed hierarchical regression analyses and we also computed a multiple mediation model to examine whether mindful awareness and encoding style fully mediated the relationship between SITUT frequency and psychological distress.

As there currently exists no validated instrument in French language to assess the general extent to which individuals experience SITUTs in daily life, we dedicated a first set of studies to the validation of a French version of the Daydreaming Frequency Scale (DDFS; Giambra, 1993). This self-report questionnaire is currently the most widely used retrospective measure of mind-wandering and daydreams. It is sensitive to the effect of aging on SITUT frequency (Giambra, 1993) and is related to depressive symptomatology (Giambra and Traynor, 1978), mindful awareness, and the frequency of task-unrelated thoughts probed during mindful breathing tasks (Mrazek et al., 2012). In Study 1A, we investigated the factorial structure of the French version of the questionnaire using a principal component analysis. We also examined whether scores on the DDFS were related to age, to the general tendency to experience positive and negative affect in daily life, and to the frequency and clarity with which individuals see themselves in the future. In Study 1B, we performed a confirmatory factor analysis (CFA) to further examine the factorial structure of the DDFS. In addition, we investigated whether the DDFS was related to measures of anxiety and depression and, to ensure that the validity of the scale is not excessively flawed by its retrospective nature, we also examined whether DDFS scores are related to an online measure of SITUTs sampled during an attentional laboratory task, the Sustained Attention to Response Task (SART; Robertson et al., 1997). Then, in Study 2, we used the validated adaptation of the DDFS to examine whether SITUT frequency still accounts for psychological distress after controlling for individual differences in the tendency to pay attention to the present-moment.

## Study 1A

In this study, we first examined the factorial structure of the French translation of the DDFS using a principal component analysis. Next, we examined whether scores on this scale were related to participants’ age and to the general experience of negative and positive affect in daily life. In light of previous research, we expected that the rate of SITUTs reported on the DDFS would decrease with age (Giambra, 1989, 1993, 2000; Jackson and Balota, 2012) and would be associated with negative affect (Giambra and Traynor, 1978; Killingsworth and Gilbert, 2010). We also aimed at exploring the previously documented relationship between mind-wandering and self-related future thoughts (Smallwood et al., 2009b, 2011; Baird et al., 2011; Stawarczyk et al., 2011a). Recent findings suggest that thinking about future selves can be dissociated in two components: (i) the frequency with which people spontaneously think about themselves in the future, named “Frequency,” and (ii) the vividness with which they “see” themselves in the future, named “Clarity” (McElwee and Haugh, 2010). As previous studies have found that most SITUTs are self-related and oriented toward the future, we expected that scores on the DDFS would be related to the frequency of future self thoughts. The two dimensions of thinking about oneself in the future were assessed with a French adaptation of the Future Self Thoughts questionnaire (FST; McElwee and Haugh, 2010) that was created for the purpose of the present study.

### Methods

#### Participants

A total of 100 native French-speaking individuals (42 men) from the Belgian general population volunteered to participate in the study (see Table 1, sample A, for age and years of education).

**Table 1:** **Means, standard deviations, and ranges for the different variables assessed in samples A, B, and C**.

Variable	Mean (standard deviation)	Range
**SAMPLE A (N = 100)**		
Age	31.59 (11.52)	18–58
Achieved years of education	14.18 (2.16)	9–19
DDFS	39.41 (9.76)	18–58
FST frequency	19.42 (6.27)	6–36
FST clarity	18.40 (5.47)	5–30
PANAS positive affect	33.06 (5.38)	15–44
PANAS negative affect	22.64 (5.59)	11–40
**SAMPLE B (N = 64)**		
Age	22.50 (2.07)	19–26
Achieved years of education	14.67 (1.83)	11–18
% On-task reports	41.82 (20.73)	3.33–96.67
% TRI reports	24.32 (11.65)	3.33–56.67
% ED reports	15.21 (9.27)	0–40
% SITUT reports	18.65 (17.10)	0–76.67
DDFS	43.47 (7.75)	25–60
CES-D	15.09 (8.79)	2–43
BAI	8.58 (5.86)	0–29
**SAMPLE C (N = 100)**		
Age	22.73 (3.23)	18–30
Achieved years of education	14.25 (2.01)	9–21
DDFS	37.69 (9.20)	17–58
MAAS	63.90 (10.29)	37–88
ESQ	16.86 (5.40)	6–28
PHQ-4	2.57 (2.64)	0–11

*DDFS, Daydreaming Frequency Scale; FTS, Future Self Thoughts questionnaire; PANAS, Positive and Negative Affect Schedule (trait form);% On-task reports, percentage of on-task reports made to the thought-probes during the Sustained Attention to Response Task (SART);% TRI reports, percentage of task-related inference reports;% ED reports, percentage of external distraction reports;% SITUT reports, percentage of stimulus-independent, and task-unrelated thought reports; CES-D, Center for Epidemiological Studies-Depression scale; BAI, Beck Anxiety Inventory; MAAS, Mindful Attention Awareness Scale; ESQ, Internal and External Encoding Style Questionnaire; PHQ-4, Patient Health Questionnaire-4*.

#### Questionnaires

##### Daydreaming frequency scale

The DDFS is one of the 28 scales composing the Imaginal Process Inventory, a 344 item questionnaire designed for in-depth assessment of individuals’ inner mental life (Singer and Antrobus, 1963, 1970, 1972). The French version of the DDFS consists of 12 items adapted from the original English version of the questionnaire (Giambra, 1993), and was developed using the back-translation method (e.g., Carlson, 2000). The 12 items of the original DDFS were first translated into French by two of the authors (David Stawarczyk and Arnaud D’Argembeau). Another independent translator then translated the French version back into English. The original version and the back-translation of the DDFS were compared, and the discrepancies between these two English versions were discussed until a satisfactory solution was found by the different translators, and the French version was modified accordingly. The translation of the questionnaire is presented in Appendix A. Respondents are asked to rate the extent to which they experience daydreaming in their daily life with reference to a five-point Likert-scale, ranging from “A” to “E.” The wording of the response options to which the letters correspond differs among the items but the higher placed letters in the alphabetical order always correspond to an increased experience of SITUTs in daily life. Values of 1, 2, 3, 4, or 5 were assigned to the options depending on their ordinal position on the continuum. Previous studies have demonstrated the good psychometric properties of the original DDFS. For instance, an exploratory factor analysis performed on the 28 scales of the Imaginal Process Inventory completed by 1353 adults from the general population demonstrated that each of the 12 items of the DDFS loaded on a single exclusive factor with minimum values of 0.50 (Giambra, 1980). Other analyses demonstrated that the internal consistency reliability (Cronbach’s alpha) of the original DDFS was 0.91 and its test-retest reliability was 0.76 for an interval of 1 year or less (Giambra, 1993).

##### Future self thoughts questionnaire

The FST (McElwee and Haugh, 2010) consists of 11 items designed to assess two dimensions of thoughts about one’s future selves: (i) Frequency, the extent to which respondents spontaneously think of themselves in the future (items 2, 4, 6, 7, 8, 11), and (ii) Clarity, the vividness with which respondents “see” themselves in the future (items 1, 3, 5, 9, 10). Respondents are asked to rate the extent to which each statement describes how they think or act in their daily life with reference to a six-point Likert-scale ranging from 1 (*not at all true for me*) to 6 (*completely true for me*). Items 1, 3, 5, and 10 are reverse-scored. Cronbach’s alphas of the Frequency and Clarity scales in the original validation study of the FST were respectively 0.79 and 0.86. The French version of the FST was developed using the same back-translation method as described above for the DDFS. The translation of the questionnaire is presented in Appendix B.

##### The positive and negative affect schedule

The Positive and Negative Affect Schedule (PANAS) consists of two 10-item mood scales that respectively measure positive and negative affect, both as states and traits (Watson et al., 1988). Respondents are asked to rate the extent to which they experience particular emotions with reference to a five-point Likert-scale ranging from 1 (*very slightly or not at all*) to 5 (*very much*). Only the trait form was used in the present study (French version by Vautier and Raufaste, 2003): participants were asked to rate the extent to which they experience each emotion in general. Cronbach’s alphas of the positive affect and negative affect scales were respectively 0.88 and 0.87 in the original validation study of the PANAS; the corresponding values were 0.77 and 0.74 in the present study.

#### Procedure

Participants from sample A were tested individually. Each participant provided written informed consent and was first asked for demographic information before completing the questionnaires in the following order: DDFS, FST, and PANAS. This study and the other studies reported in this paper were part of a broader research project that was approved by the Ethical Committee of the faculty of Psychology and Education of the University of Liège.

### Results

#### Psychometric properties of the French version of the DDFS

**Figure 1:**
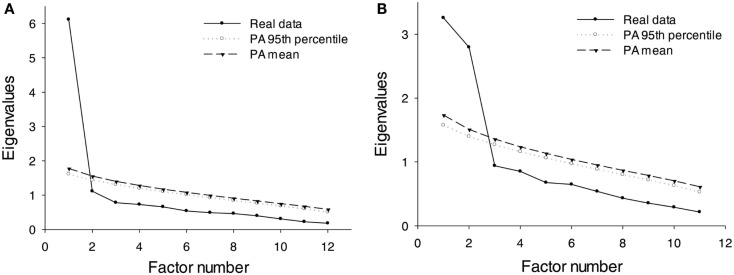
**Scree plots for the principal component analyses**. **(A,B)** respectively represent the eigenvalues for the principal component analyses performed on the DDFS and FST items for the real data sets, as well as the mean eigenvalues and upper 95th percentiles for the same analyses performed on 5000 random data sets that were obtained by permutations of the real data following Castellan’s algorithm (Castellan, 1992). PA, parallel analysis. *N* = 100 (sample A).

The factorial structure of the French version of the DDFS was analyzed using a principal component analysis. We conducted a parallel analysis (Horn, 1965; O’Connor, 2000) to determine the number of factors to be extracted. In the parallel analysis method, random data sets are generated by permutations of the raw data set, and the eigenvalues from the real data are compared to the eigenvalues from the random data. The number of factors to be extracted corresponds to the number of eigenvalues that explains more variance in the real data than in the random data (Reise et al., 2000; Hayton et al., 2004). The resulting eigenvalues for the random data are shown in Figure 1A, with the eigenvalues from the real data, which clearly indicates that a single-factor should be retained. The resulting single-component solution is presented in Table 2 with the individual component loadings for the variables included. The internal reliability of the scale was estimated using Cronbach’s Alpha value; values greater than 0.70 are generally considered acceptable (Bland and Altman, 1997). The Cronbach’s Alpha value for the French version of the DDFS was 0.91, indicating that the internal reliability of the scale is very good.

**Table 2:** **Pattern matrix factor loadings for the principal component analysis of the DDFS items (*N* = 100, sample A)**.

DDFS items (item #)	Factor 1
(Variance explained)	50.95%
• Daydreams and fantasies make up… (2)	**0.83**
• I daydream… (1)	**0.83**
• Whenever I have time on my hands, I daydream… (10)	**0.80**
• As regards daydreaming, I would characterize myself as someone who… (3)	**0.79**
• I lose myself in active daydreaming (9)	**0.73**
• When I am not paying attention to some job, book, or TV, I tend to be daydreaming… (5)	**0.70**
• I daydream at work (or school)… (7)	**0.69**
• When I am at a meeting or show that is not very interesting, I daydream rather than pay attention… (11)	**0.67**
• Recalling things from the past, thinking of the future, or imagining unusual kinds of event occupies… (8)	**0.66**
• Instead of noticing events or people in the world around me, I will spend approximately… (6)	**0.61**
• On a long bus, train, or airplane ride I daydream… (12)	**0.61**
• I recall or think over my daydreams… (4)	**0.59**

*Factor loadings exceeding 0.4 are highlighted*.

#### Psychometric properties of the FST

The factorial structure of the French translation of the FST was also analyzed with a principal component analysis. Horn’s parallel analysis showed that the two first eigenvalues from the real data were higher than those in the random data indicating a two component solution, as shown in Figure 1B. The resulting two component solution using a Varimax normalized rotation is presented in Table 3 with the individual component loading for the variables included. An orthogonal method of rotation was used as the two dimensions of future self-thought are theoretically conceived as being unrelated (McElwee and Haugh, 2010). Factor 1 corresponds to the Frequency scale of the original version and Factor 2 to the Clarity scale. All items clearly loaded onto only one-factor with the exception of Item 6 “When I daydream, I often see myself as I may be in the future,” with a loading of 0.62 onto Factor 1 and a loading of 0.46 onto Factor 2. Because this item is more consistent with Factor 1 (frequency) both empirically and conceptually, we retained it in this factor for subsequent analyses. Cronbach’s Alpha values were 0.79 for Factor 1 and 0.77 for Factor 2, indicating that the internal reliability of both scales is satisfactory.

**Table 3:** **Pattern matrix factor loadings for the principal component analysis of the FST items (*N* = 100, sample A)**.

FST items (item #)	Factor 1	Factor 2
(Variance explained)	29.60%	25.42%
• My thoughts tend to wander toward imagining possible futures for myself. (8)	**0.88**	−0.01
• It is common for me to spend time thinking about myself as I might be in future stages of life. (7)	**0.87**	0.10
• I tend to think about myself as I might be in the future even when I don’t want to be thinking about it. (11)	**0.69**	−0.02
• When I daydream, I often see myself as I may be in the future. (6)	**0.62**	**0.46**
• Thinking about myself in the future often makes me have strong feelings (whether happy or sad). (4)	**0.58**	−0.16
• I often picture myself in the future in different ways and think about the various paths that could lead me to those different futures. (2)	**0.49**	−0.07
• Images of myself in the future are very “hazy,” not clear at all.* (5)	−0.08	**0.79**
• My future seems vague and uncertain to me. * (1)	−0.16	**0.77**
• When I picture myself in the future, I see clear and vivid images. (9)	0.33	**0.69**
• I really find it hard to predict what I might be like in the future.* (3)	0.10	**0.68**
• My future is too uncertain for me to plan very far ahead.* (10)	−0.27	**0.66**

Factor loadings exceeding 0.4 are highlighted. *: reverse-scored items.

#### Correlational analyses

Means and standard deviations for age, number of achieved years of education, scores on the positive, and negative affect scales of the PANAS, as well as the Clarity and Frequency scales of the FST, and the DDFS are presented in Table 1 (sample A). Correlations between the variables are presented in Table 4. Results of the correlation matrix including all the variables showed a positive relationship between the DDFS and the Frequency scale of the FST. This result indicates that participants who reported to experience more SITUTs in their daily life also reported more spontaneous thoughts about their future self. As expected, scores on the DDFS were also positively related to the negative affect scale of the PANAS and were negatively related to age, indicating that participants with higher rates of SITUTs were younger and generally experienced more negative emotions in daily life. It is also worth noting that the Clarity scale of the FST was not significantly related to SITUT frequency. Next, we performed partial correlation analyses (see Table 4) to examine whether the relationships between the different scales remained significant after controlling for age and educational level. Results showed that the correlations between the DDFS and FST Frequency, and between the DDFS and negative affect, remained significant after partialing out the influence of age and educational level. Finally, we also checked for a possible effect of gender on these results. Mean comparisons showed no gender difference for the different scales, and controlling for gender did not influence the significance of the correlation analyses.

**Table 4:** **Correlation matrices of study 1A variables (*N* = 100, sample A)**.

	1	2	3	4	5	6	7
Age		–	–	–	–	–	–
Educ.	0.06		–	–	–	–	–
DDFS	−0.32**	−0.01		0.36**	−0.13	−0.02	0.20*
FST freq.	−0.38**	0.07	0.43**		0.09	−0.09	0.20
FST clar.	0.09	0.27**	−0.14	0.07		0.25*	−0.02
PANAS pos.	0.06	0.09	−0.03	−0.10	0.27**		−0.01
PANAS neg.	−0.11	−0.27**	0.21*	0.19	−0.10	−0.04	

Educ., number of achieved years of education; DDFS, Daydreaming Frequency Scale; FTS freq., frequency scale of the Future Self Thoughts questionnaire; FST clar., clarity scale of the Future Self Thoughts questionnaire; PANAS pos., positive affect scale of the Positive and Negative Affect Schedule (trait form); PANAS neg., negative affect scale of the Positive and Negative Affect Schedule (trait form). Values below the diagonal are the correlations between all the variables; Values above the diagonal are the correlations between the variables after controlling for Age and Educ.; *significant at *p *< 0.05 (two-tailed); **significant at *p *< 0.01 (two-tailed).

### Discussion

This first study showed that the French translation of the DDFS has a single-factor structure and good internal reliability. In addition, we demonstrated that scores on this scale are negatively related to age, which is concordant with the previous findings that the frequency of SITUTs decline in aging (Giambra, 1989, 1993, 2000; Jackson and Balota, 2012). We also found that the frequency of SITUTs is related to negative affect, which is also in line with previous findings (Giambra and Traynor, 1978; Killingsworth and Gilbert, 2010). Finally, our results provide additional specification of the previously observed relationship between mind-wandering and future thinking. On the one hand, the finding that the Frequency dimension of the FST correlates with the DDFS is consistent with previous experience sampling studies which showed that imagining oneself in the future represents a substantial part of the content of SITUTs probed during cognitive tasks (Smallwood et al., 2009b, 2011; Baird et al., 2011; Stawarczyk et al., 2011a). Interestingly, however, we found that the Clarity dimension of the FST was unrelated to the self-reported tendency to experience SITUTs in daily life. These results suggest that although SITUTs are often self-related and future-oriented, they do not necessarily feature clear and vivid images of the self in the future.

## Study 1B

Study 1A showed that the French version of the DDFS has a single-factor structure. In Study 1B, we further tested the validity of this single-factor structure with a CFA. Additionally, we examined whether the retrospective measure of SITUT frequency in daily life given by the DDFS is related to the frequency of SITUTs sampled during the SART (Robertson et al., 1997). The purposes of this analysis were twofold. First, we wanted to ensure that the estimate of SITUT frequency provided by the DDFS is not excessively flawed by the retrospective nature of this instrument; we thus examined whether the scores on the DDFS correlate with an “online” measure of SITUTs. Second, we wanted to examine the specificity of the DDFS as a measure of SITUTs in comparison to other kinds of conscious experiences that can occur when one’s mind is not fully focused on the task at hand, such as thoughts related to the appraisal of the task (i.e., task-related interferences) and distractions by task-unrelated exteroceptive perceptions and interoceptive sensations (i.e., external distractions). To do so, we used a newly validated experience sampling method which permits to clearly distinguish SITUTs from other kinds of conscious experiences during laboratory task performance (Stawarczyk et al., 2011a,b). We expected that DDFS scores would be related to the frequency of SITUTs during the SART, but not to the frequency of task-related interferences and external distractions. Finally, besides the online sampling of SITUTs, Study 1B also included questionnaire measures of depressive and anxious symptomatology to further document the relationship between psychological well-being and SITUTs revealed in Study 1A.

### Methods

#### Participants

A total of 164 native French-speaking individuals (54 men) from the Belgian and Swiss general populations volunteered to participate in Study 1B (samples B and C, see Table 1) and were included in the CFA. Other analyses of Study 1B were conducted on 64 of these participants (31 men; sample B).

#### Questionnaires and task

##### Daydreaming frequency scale

See the Methods Section of “Study 1A” for a detailed description of this scale. Cronbach’s alpha for the DDFS was 0.88 in the present study.

##### Center for epidemiological studies-depression scale

The Center for Epidemiological Studies-Depression scale (CES-D) is used to assess depressive symptomatology in non-clinical populations. It comprises 20 items assessing the presence of depressive symptoms in the past week with reference to a four-point Likert-scale ranging from 0 (*never, rarely: less than 1 day*) to 3 (*frequently, all the time: between 5 and 7 days*). Items 4, 8, 12, and 16 are reverse-scored (original version, Radloff, 1977; French version, Fuhrer and Rouillon, 1989). Cronbach’s alpha for the CES-D was 0.85 in the original validation study of the scale and was 0.89 in the present study.

##### Beck anxiety inventory

The Beck Anxiety Inventory (BAI; original version, Beck et al., 1988; French version, Freeston et al., 1994) is used to assess anxiety in adults during the last 7 days and comprises 21 items. Respondents are asked to rate how much they have been affected by certain anxiety symptoms (emotional, physiological, and cognitive) in the past week on a four-point Likert-scale ranging from 0 (*not at all*) to 3 (*severely*). Cronbach’s alpha for the BAI was 0.92 in the original validation study of the scale and was 0.78 in the present study.

##### Sustained attention to response task with thought-probes

The version of the SART used in this study is similar to the one used in Stawarczyk et al. (2011a). Stimuli (numbers between 1 and 9) were presented sequentially at the center of the screen. Participants were asked to respond as fast and accurately as possible to the numbers and to withhold their response when presented with the number 3 (the target stimulus). The probability of the target stimulus was 11%. The interstimulus interval was 2000 ms, and the duration of each stimulus (target and non-targets) was 500 ms. The task comprised 30 blocks whose duration was either 25, 35, 45, 55, or 65 s. Each block was immediately followed by a thought-probe which interrupted the task. For each probe, participants were asked to characterize the ongoing conscious experience they had just prior to the probe. Four possible choices were provided: (i) on-task reports: the participant’s attention and thoughts were fully focused on the task-related stimuli; (ii) task-related interferences reports: the participant experienced thoughts about the task that did not help him/her to have the best possible performance on the current ongoing trials (e.g., thoughts about task duration or about the participant’s overall performance); (iii) external distractions reports: the participant’s attention was focused on stimuli that were present in the current environment but unrelated to the task at hand (e.g., exteroceptive perceptions or interoceptive sensations); and (iv) mind-wandering reports: the participant had his/her attention decoupled from the external environment and was experiencing thoughts unrelated to the task at hand (e.g., thoughts about what the participant did last evening). In addition to the thought-probes, participants were asked directly after the SART to complete the thinking content component of the Dundee Stress State Questionnaire (Matthews et al., 1999), which retrospectively assessed the frequency with which they experienced SITUTs and task-related interferences during the SART. The scale did not show satisfactory psychometric properties in the present sample, however, and will not be analyzed further.

#### Procedure

All participants from samples B and C were tested individually and provided written informed consent. Each participant was asked for demographic information at the beginning of the testing session. Participants from sample B completed the SART before the questionnaires, which were administered in the following order: DDFS, CES-D, and BAI. Participants from sample C completed the questionnaires in the following order: DDFS, Internal and External Encoding Style Questionnaire (ESQ), MAAS, and Patient health questionnaire-4 (PHQ-4; see Study 2 for more detail about the latter three instruments). In addition to the measures reported here, participants from Sample B and C completed a series of cognitive tasks (assessing attentional control abilities) at the beginning of the testing session. These tasks were not relevant to the aims of Studies 1B and 2 and will not be discussed further here.

### Results

#### Confirmatory factor analysis of the DDFS

The one-factor structure found in Study 1A for the DDFS was tested via a CFA computed with Lisrel 8.8 (Jöreskog and Sörbom, 1996) and performed on the total number of participants from samples B and C (i.e., 164 participants). The maximum likelihood method was performed on the covariance matrix of the DDFS raw scores for each item. Goodness of fit was tested with the χ^2^ (a non-significant value corresponds to an acceptable fit). The χ^2^ is known to increase with sample size, however, and it has been emphasized that it is unusual to obtain a non-significant χ^2^ when performing CFA on self-report questionnaires (Byrne, 1994). Therefore, the model fit was assessed by determining whether the observed χ^2^ value was less than three times the model degrees of freedom (Schermelleh-Engel et al., 2003; Iacobucci, 2010). In addition, three indices of model fit were computed: the Root Mean Square Error of Approximation (RMSEA), the Standardized Root Mean Square Residual (SRMR), and the Comparative Fit Index (CFI). RMSEA and SRMR values respectively below 0.08 and 0.10 represent an acceptable fit of the model, and the lower the better (Schermelleh-Engel et al., 2003). CFI values above 0.95 represent an acceptable fit (values closer to 1.00 represent better fit; Schermelleh-Engel et al., 2003; Hooper et al., 2008). Results indicated that the χ^2^ statistic for the model was significant χ^2^ (54) = 107.685, *p *< 0.001, and the χ^2^/*df* value was 1.994. In addition, we obtained a RMSEA = 0.078, a SRMR = 0.046, and a CFI = 0.979. The combination of these four indices indicated an acceptable fit.

#### The DDFS, online measures of SITUTs, and levels of anxious and depressive symptoms

Means and standard deviations for the proportions of each of the four kinds of conscious experiences sampled during the SART (being fully focused on-task, task-related interferences, external distractions, and SITUTs), as well as DDFS, CES-D, and BAI scores are presented in Table 1 (sample B). Correlation analyses between the different variables are presented in Table 5. The results mainly showed that participants who scored higher on the DDFS reported more mind-wandering episodes and made fewer reports of being fully focused on-task during the SART. On the other hand, reports of task-related interferences and external distractions during the SART were unrelated to the DDFS scores. Regarding the CES-D and BAI, we found that a higher self-reported frequency of SITUTs in daily life was related to higher levels of anxiety and depressive symptoms during the past week. We also checked for the presence of gender effects in this study. Mean comparisons only showed a significant effect of gender for the percentage of external distraction reports made during the SART, with women reporting more external distractions than men [*t*(62) = 2.27; *p* = 0.03; women = 17.68 ± 9.74; men = 12.58 ± 8.11]; controlling for gender did not influence the significance of the correlation analyses.

**Table 5:** **Correlation matrix of Study 1B variables (*N* = 64, sample B)**.

	% On-task	% TRIs	% EDs	% MW	DDFS	CES-D
DDFS	−0.34**	0.11	0.07	0.30*		
CES-D	−0.22	0.22	−0.18	0.22	0.37**	
BAI	−0.20	0.28*	0.20	−0.05	0.28*	0.33**

On-task, percentage of on-task reports made to the thought-probes during the Sustained Attention to Response Task (SART); TRIs, percentage of task-related inference reports made to the thought-probes during the SART; EDs, percentage of external distraction reports made to the thought-probes during the SART; MW, percentage of mind-wandering reports made to the thought-probes during the SART; DDFS, Daydreaming Frequency Scale; CES-D, Center for Epidemiological Studies-Depression scale; BAI, Beck Anxiety Inventory. *Significant at *p *< 0.05 (two-tailed); **significant at *p *< 0.01 (two-tailed).

### Discussion

In Study 1B, we validated the single-factor structure of the French translation of the DDFS with a CFA. We then showed that the retrospective measure of SITUTs in daily life provided by the DDFS is related to an online measure of mind-wandering during the SART. This finding of a concordance between trait and state measures of SITUTs indicates that the validity of the DDFS is not excessively flawed by the retrospective nature of this questionnaire. Moreover, scores on the DDFS were unrelated to other kinds of conscious experiences that occur when one’s mind is not fully focused on-task, namely task-related interferences, and external distractions (Stawarczyk et al., 2011a,b). These results demonstrate the specificity of the DDFS as a measure of SITUTs relative to the other kinds of distracting conscious experiences investigated here during the SART. Finally, we found that SITUT frequency was related to depressive and anxious symptomatology, confirming that mind-wandering is related to lower psychological well-being (Giambra and Traynor, 1978; Killingsworth and Gilbert, 2010; Burg and Michalak, 2011).

## Study 2

Having established the validity of the DDFS as a self-report measure of the general frequency of SITUTs in daily life, we aimed in Study 2 to examine whether the relationship between mind-wandering and lower psychological well-being that has been documented in previous studies, as well as in the present study, can be accounted for by the extent to which individuals tend to be generally aware of the present-moment. Numerous studies on dispositional mindfulness have found that decreased attention to the here and now is related to lower psychological well-being (Brown and Ryan, 2003; Brown et al., 2007; Jermann et al., 2009; Keng et al., 2011). Although some recent findings have revealed that SITUT frequency is related to lower mindful awareness (Burg and Michalak, 2011; Mrazek et al., 2012), no study to date has examined whether individual differences in present-moment attention can account for the relationship between SITUT frequency and psychological distress.

Here, we tested this hypothesis by using two different self-report measures indicative of present-moment attention in daily life. The first measure was the Mindful Attention Awareness Scale (MAAS), a single-dimension scale specifically designed to assess the frequency of open and receptive attention to, and awareness of ongoing events and experiences during everyday activities (MAAS; Brown and Ryan, 2003; Jermann et al., 2009). The second measure was encoding style, as indexed by the ESQ (Lewicki, 2005; Billieux et al., 2009). The ESQ is based on the assumption that the perception of environmental objects involves both encoding stimuli (perceptual features) from the environment and retrieving concepts (schemata) from long-term memory that fit these stimuli. Lewicki (2005) further proposes that there are individual differences in the “threshold of instantiation of schemata.” For this author, there is a continuum between “external encoders” who collect a relatively high amount of supportive evidence from the external environment before imposing an interpretative category (schema) on a stimulus and “internal encoders” who are comparatively less carefully attending to the external environment and hastily interpret situations in terms of preexisting (internal) encoding categories. The ESQ characterizes individuals along this continuum and therefore reflects individual differences in the attendance to the external environment versus internal mental processes.

There is growing evidence that being receptive to present events and experience contributes to well-being and happiness (for reviews, see Brown et al., 2007; Keng et al., 2011) and people who present an internal encoding style and who are less mindful indeed report lower psychological well-being and higher levels of emotional disturbance, such as depression and anxiety (Brown and Ryan, 2003; Lewicki, 2005; Jermann et al., 2009). Therefore, it could be that the relationship between mind-wandering and psychological distress that has been documented in previous studies is not due to SITUT frequency *per se* but rather to the fact that people who experience more mind-wandering tend to be less aware of the present-moment. To test this hypothesis, we performed regression and mediation analyses to investigate whether SITUT frequency explains a significant part of the variance of psychological distress beyond what is explained by mindful awareness and encoding style.

### Methods

#### Participants

A total of 100 native French-speaking individuals (23 men) from the Swiss general population volunteered to participate in the study (see Table 1, sample C).

#### Questionnaires

##### Daydreaming frequency scale

See the Methods Section of “Study 1A” for a detailed description of this scale. Cronbach’s alpha for the DDFS was 0.92 in the present study.

##### Mindful attention awareness scale

The MAAS is a 15-item scale measuring the general tendency to be attentive to and aware of present-moment experience in daily life (Brown and Ryan, 2003). Using a six-point Likert-scale ranging from 1 (*almost always*) to 6 (*almost never*), respondents rate how often they have experiences of acting on automatic pilot, being absorbed in one’s thoughts and emotions, and not paying attention to ongoing sensory information (e.g., “I find myself listening to someone with one ear, doing something else at the same time” and “I tend to walk quickly to get where I’m going without paying attention to what I experience along the way”). Higher scores indicate a general tendency to be more aware of and attentive to one’s current experience, including perceptual information, as well as one’s thoughts, emotions, and overt behavior (French version, Jermann et al., 2009). Cronbach’s alpha for the MAAS varied between 0.80 and 0.87 in the original validation study of the scale and was 0.84 in the present study.

##### Internal and external encoding style questionnaire

The ESQ was designed to assess individual differences in the tendency to rely on information coming directly from the senses versus on preexisting, internal schemata in the process of perception (Lewicki, 2005). Using a six-point Likert-scale ranging from 1 (*strongly disagree*) to 6 (*strongly agree*), respondents rate the frequency with which they experience “split-second illusions” in daily life, such as seeing (erroneously) an animal running across the road, only to find out a moment later that it was a piece of paper or a leaf blown by the wind. The scale consists of 21 items, but only six items (number 5, 8, 11, 15, 18, and 21) are diagnostic items; the remaining 15 items are included to conceal the focus of the test. Lower scores on the ESQ are indicative of an “external encoding style,” meaning that the individual generally pays close attention to what is happening in the external environment, while a high score indicates an “internal encoding style,” implying that the individual is less attentive to incoming sensory information and relies instead more on his or her own expectations when encoding external stimuli. No Cronbach’s alpha was provided by Lewicki (2005) for the original version of the ESQ. It was 0.77 in the validation study of the French version of the scale (Billieux et al., 2009), and 0.69 in the present study.

##### Patient health questionnaire-4

The PHQ-4 is a brief self-report questionnaire of four items designed to assess depression and anxiety (Kroenke et al., 2009). The questionnaire begins with the stem question: “Over the last 2 weeks, how often have you been bothered by the following problems?” Respondents are asked to rate each statement with reference to a four-point Likert-scale ranging from 0 (*not at all*) to 3 (*nearly every day*). Statements referring to anxiety are “Feeling nervous, anxious, or on the edge” and “Not being able to stop or control worrying.” Statements referring to depression are “Feeling down, depressed or hopeless” and “Little interest or pleasure in doing things.” This questionnaire was originally created to provide separate scores for depression and anxiety but subsequent psychometric analyses have shown that it can also be used as a general marker of psychological distress with a single aggregated score (Lowe et al., 2010). Higher total scores on the PHQ-4 indicate higher levels of psychological distress and are predictive of functional impairment, disability days, healthcare use, as well as lower self-esteem, life satisfaction, and resilience (Kroenke et al., 2009; Lowe et al., 2010). Given that DDFS scores were related to both depressive and anxious symptomatology in Study 1B and that the scores for the anxiety and depression items of the PHQ-4 were strongly correlated in the present study (*r* = 0.66, *p *< 0.01), only the total score of the PHQ-4 was used in subsequent analyses. Cronbach’s alpha for the total score of the PHQ-4 was 0.82 in the original validation study of the instrument and was 0.83 in the present study.

#### Procedure

See the Procedure Section of “Study 1B” for a detailed description of the procedure used with sample C.

### Results

#### Relationships between mind-wandering, mindful awareness, encoding style, and psychological distress

Means and standard deviations for the DDFS, MAAS, ESQ, and PHQ-4 scores are presented in Table 1 (sample C). Correlation analyses between the different variables are presented in Table 6. As expected, these analyses demonstrated that individuals who reported to experience SITUTs more frequently in their daily life had the propensity to be less mindful of the present-moment, presented a more internally oriented encoding style, and reported higher psychological distress. In addition, we found that psychological distress, mindful awareness, and internal encoding style were all intercorrelated.

**Table 6:** **Correlation matrix of Study 2 scales (*N* = 100, sample C)**.

	1	2	3
DDFS			
MAAS	−0.37**		
ESQ	0.26*	−0.39**	
PHQ-4	0.22*	−0.43**	0.38**

DDFS, Daydreaming Frequency Scale; MAAS, Mindful Attention Awareness Scale; ESQ, Internal and External Encoding Style Questionnaire; PHQ-4, Patient Health Questionnaire-4. *Significant at *p *< 0.05 (two-tailed); **significant at *p *< 0.01 (two-tailed).

A set of hierarchical multiple regression analyses were performed to examine whether the relationship between the DDFS and PHQ-4 can be accounted for by the MAAS and ESQ. DDFS scores on one hand and MAAS together with ESQ scores on the other hand were entered in alternating order at Steps 1 and 2. As can be seen in Table 7, the final regression model accounted for a significant proportion of variance of the PHQ-4 (*R*^2^ = 0.24, *p *< 0.01). Furthermore, these analyses showed that both the MAAS and ESQ remained significant predictors of the PHQ-4 beyond the DDFS, whereas the DDFS did not remain a significant predictor of the PHQ-4 once the MAAS and ESQ were entered into the regression model. These results indicate (i) that the relationship between SITUT frequency and psychological distress can be accounted for by the extent to which individuals tend to be aware of the present-moment, (ii) that the relationship between measures of present-moment attention and psychological distress cannot be explained by SITUT frequency, and (iii) that mindful awareness as well as encoding style each explain an independent part of the variance of psychological distress.

**Table 7:** **Hierarchical multiple regression analyses of Study 2 scales (*N* = 100, sample C)**.

Predictor	Δ*R*^2^	Adjusted Δ*R*^2^	Standardized β
1. DDFS	0.05	0.04	0.22*
2. MAAS			−0.32**
2. ESQ	0.19	0.17	0.25*
1. MAAS			−0.33**
1. ESQ	0.22	0.20	0.25*
2. DDFS	<0.01	<0.01	0.04

DDFS, Daydreaming Frequency Scale; MAAS, Mindful Attention Awareness Scale; ESQ, Internal and External Encoding Style Questionnaire; PHQ-4, Patient Health Questionnaire-4. *Significant at *p *< 0.05 (two-tailed); **significant at *p *< 0.01 (two-tailed).

**Figure 2:**
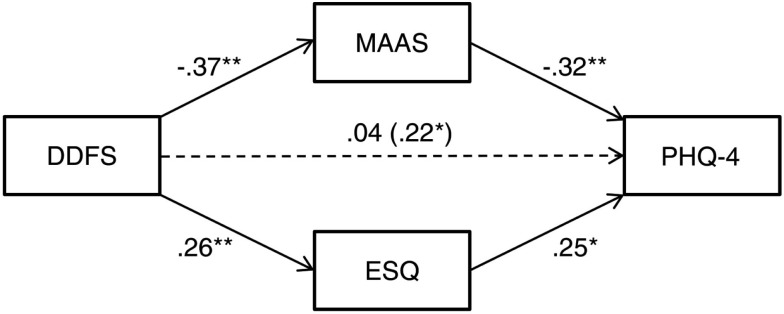
**Multiple mediation model of Study 2 scales**. DDFS, Daydreaming Frequency Scale; MAAS, Mindful Attention Awareness Scale; ESQ, Internal and External Encoding Style Questionnaire; PHQ-4, Patient Health Questionnaire-4. Values next to each line represent the standardized coefficient of each path. Value between rows represents the total effect of the DDFS on the PHQ-4. *Significant at *p *< 0.05 (two-tailed); **significant at *p *< 0.01 (two-tailed).

Although this study was correlational in nature and did not use an experimental design, we nonetheless computed a multiple mediation model (Preacher and Hayes, 2008) to examine whether and how the ESQ and MAAS mediate the relationship between the DDFS and PHQ-4. A bootstrapping procedure based on 5000 resamples was used to calculate a 95% bias corrected confidence interval (BCCI) around the total indirect effect. Values for this BCCI were (0.088; 0.302); zero falling outside this interval indicates that the total mediation effect was significant. As illustrated in Figure 2, the direct effect of the DDFS on the PHQ-4 was not significant once the MAAS and ESQ were taken into account as mediating variables (β* *= 0.04, *p *= 0.66). A similar procedure was used to calculate the specific indirect effect of the MAAS and ESQ, which were respectively (0.029; 0.248) and (0.002; 0.169). These results indicate that each of these scales was a significant intervening variable in the mediation model beyond and above the other one. A contrast analysis showed that the strength of the mediating effect of the ESQ and the MAAS did not differ from each other, with a BCCI of (−0.088; 0.243).

Finally, we also checked for the presence of gender effect in this study. Mean comparisons showed a significant effect of gender only for the DDFS, with women scoring higher than men on this scale [*t*(98) = 2.18; *p* = 0.03; women = 38.77 ± 8.68; men = 34.09 ± 10.14]; controlling for gender did not influence the significance of the correlation, regression, and mediation analyses reported above.

### Discussion

In Study 2, we further established that SITUT frequency is associated with higher psychological distress and also found, as expected, that these thoughts are related to lower mindful awareness and a more internally oriented encoding style. These latter results are concordant with the perceptual decoupling hypothesis of mind-wandering, which suggests that SITUTs consume cognitive resources and thus are associated with reduced processing of ongoing perceptual information (Smallwood, 2010, 2011; Schooler et al., 2011). More importantly, we found that both internal encoding style and lower mindful awareness fully accounted for the relationship between SITUT frequency and psychological distress. Indeed, the effect size of this relationship became almost null (standardized coefficient of 0.04) when mindful awareness and encoding style were taken into account into the regression model, and a multiple mediation model confirmed that the MAAS and ESQ fully mediated the relationship between SITUT frequency and psychological distress. On the other hand, both mindful awareness and encoding style independently explained psychological distress beyond what was already explained by SITUT frequency. These results suggest (i) that it may not be SITUT frequency *per se* that induces higher psychological distress but rather the extent to which individuals tend to be unaware of the present-moment, (ii) that factors other than SITUT frequency are responsible for the relationship between the propensity to be aware of the present-moment and psychological distress, and (iii) that some of these factors are specific to mindful awareness and internally oriented encoding style. These three points will be discussed further in the Section “General discussion.”

## General Discussion

In three studies, we first validated a French translation of the DDFS (Giambra, 1993) and determined whether this version of the DDFS is associated with related constructs within the general framework of research on mind-wandering (i.e., age, future self-thought, and “online” measures of conscious experiences sampled during an attentional task). Next, we examined whether the negative relationship that SITUTs have with mood and psychological well-being (Killingsworth and Gilbert, 2010) is due to SITUT frequency *per se* or rather to individual differences in the extent to which people pay attention to the present-moment. To our knowledge, this latter analysis constitutes the first attempt to investigate the nature of the relationships between SITUT frequency, psychological well-being, and present-moment awareness.

Our main results can be summarized as follows. First, in line with previous research (Giambra and Traynor, 1978; Smallwood et al., 2007, 2009a; Killingsworth and Gilbert, 2010), we consistently found in each study that the self-reported general frequency with which people experience SITUTs in daily life is related to lower psychological well-being, as indexed by negative affect and level of psychopathological symptoms. Second, we found that SITUT frequency was related to both lesser mindful awareness of the present-moment, in keeping with previous results (Burg and Michalak, 2011; Mrazek et al., 2012), and to the tendency to present an internally oriented encoding style (Lewicki, 2005). These findings fit well with the idea that SITUTs consume processing resources and, therefore, tend to be associated with reduced attention to ongoing sensory information (Smallwood, 2010, 2011; Schooler et al., 2011). Third, we found that the relationship between SITUT frequency and psychological distress was fully accounted for by the extent to which individuals tend to be aware of the present-moment. Indeed, the strength of the association between SITUT frequency and psychological distress became almost null once the influences of mindful awareness and encoding style were taken into account. These results suggest that factors associated with decreased attention to the present-moment may be responsible for the negative relationship between SITUT frequency and psychological distress. Finally, mindful awareness and encoding style both explained an independent part of the variance of psychological distress beyond what was explained by SITUT frequency.

The latter finding suggests that mindful awareness and encoding style affect psychological well-being through at least partly distinct processes. Mindful awareness involves the ability to decenter from one’s thoughts and view them as passing mental events rather than to identify with them and believe thoughts to be accurate representations of reality (Brown et al., 2007; Keng et al., 2011). The beneficial effect of mindful awareness in terms of psychological distress might thus be due, in part, to the implementation of more effective emotion regulation strategies and a capacity to detach from maladaptive self-related thoughts (Holzel et al., 2011). A recent study using path analysis indeed showed that individual differences in the ability to regulate one’s own emotions through increased positive reappraisal (i.e., having thoughts whose purpose is to give a positive meaning to negative events in terms of personal growth) and reduced self-blame (i.e., having thoughts that blame oneself for what one has experienced) partially mediated the relationship between mindful awareness and depressive symptomatology (Jermann et al., 2009). The impact of encoding style on psychological distress might result from other mechanisms, such as “self-perpetuation” (Lewicki, 2005). It has indeed been suggested (Lewicki, 2005) that internal encoders have a tendency to persevere in erroneously interpreting stimuli on the basis of outdated or false schemata despite the repeated lack of supportive evidence from the environment (e.g., a person might continue to think he/she is particularly bad at some task despite successfully performing on several occasions). This self-perpetuation bias might lead to inflexible thinking and lower psychological well-being in a manner analogous to other well-known processes, such as arbitrary inference (coming to conclusions without sufficient environmental evidence) and overgeneralization (drawing overly broad implications from single events; Lewicki, 2005; Herndon, 2008).

In addition to these distinct processes, the present results suggest that a factor common to mindful awareness and encoding style partly underlies their relationship with psychological distress. Indeed, although both remained significant predictors, mindful awareness, and encoding style saw a decrease in the strength of their association with psychological distress when they were considered conjointly in the regression model; both constructs thus shared a part of their influence on psychological distress. This shared influence might be related to the occurrence of cognitive failures in daily life. Several studies have shown that lower mindful awareness (Cheyne et al., 2006; Carriere et al., 2008; Smilek et al., 2010) and internally oriented encoding style (Herndon, 2008) are both related to the occurrence of cognitive failures, which have in turn been related to lower psychological well-being (e.g., Mahoney et al., 1998; Wagle et al., 1999). Interestingly, Carriere et al. (2008) conducted path analyses on measures of mindful awareness, psychological distress, and cognitive failures. These authors found that the best fitting model was one in which cognitive failures partially mediated the relationship between mindful awareness and psychological distress. Thus, although this proposal remains to be investigated, it could be that the shared influence of mindful awareness and encoding style on psychological distress is related, in part, to the occurrence of cognitive failures.

Although the present results provide important insights into the nature of the relationships between SITUT frequency, present-moment awareness, and psychological well-being, several limitations have to be acknowledged. First, the present studies solely relied on retrospective self-reports of daily life experiences. Future work should assess whether similar results can be found with more ecological measures, for instance relying on experience sampling methods (e.g., Hurlburt and Akhter, 2006) that can notably provide a more direct measure of SITUT frequency in daily life. Second, the PHQ-4 is a general measure of psychological distress and more specific instruments that can discriminate between various aspects of depressive and anxious symptomatology should be used in future studies to precisely assess which aspect(s) of psychological distress are related to SITUT frequency and present-moment awareness. Third, the present work did not assess the possible influence of moderating variables in the relationship between SITUT frequency and lower psychological well-being. Repetitive SITUTs with abstract and negatively valenced content have been proposed to be unconstructive and particularly predictive of lower mood and psychological distress (Watkins, 2008, 2010). Future studies should therefore assess the content of SITUTs to determine whether experiencing constructive versus unconstructive SITUTs moderate the association between the frequency of these thoughts and psychological distress. The moderating influence of personality traits, such as psychological absorption (Tellegen and Atkinson, 1974), might also be interesting to investigate. Finally, although the multiple mediation analyses reported here suggest that the MASS and ESQ mediate the relationship between the DDFS and PHQ-4, the design of the present study was correlational and future research should employ experimental designs to assess more precisely the causal relationship between SITUTs frequency, psychological distress, and present-moment awareness.

In conclusion, this study validates a self-report instrument to assess the general frequency of SITUTs in daily life and provides preliminary evidence that the extent to which mind-wandering negatively affects psychological well-being may not be related to the occurrence of SITUTs *per se*, but could rather be explained by a general tendency to be less attentive to the present-moment. Interestingly, two different measures of present-moment attention – mindful awareness and encoding style – independently explained a part of the variance of psychological distress beyond the contribution of SITUT frequency. We have tentatively proposed that thought and emotion regulation strategies, self-perpetuation bias, and cognitive failures might be intervening processes in the relationship between psychological distress and present-moment attention. More generally, the present findings suggest that, although mind-wandering and present-moment awareness are related constructs, they are not reducible to one another, and are distinguishable in terms of their relationship with psychological well-being.

## Conflict of Interest Statement

The authors declare that the research was conducted in the absence of any commercial or financial relationships that could be construed as a potential conflict of interest.

## Footnotes

^1^It should be noted that although the constructs of mind-wandering and mindfulness are related (Mrazek et al., 2012), they are not equivalent. Mindfulness is not antithetical to thoughts, but rather fosters a different relationship to them: it entails the ability to decenter from one’s thoughts and view them as passing mental events rather than to identify with them and believe thoughts to be accurate representations of reality (Brown et al., 2007; Keng et al., 2011).

## Appendix

### A Daydreaming frequency scale

**Table:**

Nous vous demandons votre coopération pour répondre à un questionnaire portant sur votre tendance à rêvasser, à laisser votre esprit vagabonder et à être perdu dans vos pensées. Soyez attentif à bien faire la différence entre **réfléchir à ce vous êtes en train de faire** à un moment donné (par exemple, imaginer activement des solutions à un problème que vous êtes en train d’essayer de résoudre sur le moment) et **rêvasser sur quelque chose d’autre** (par exemple, penser à une prochaine sortie alors que vous essayez d’étudier). Penser à une tâche alors que vous êtes en train de la réaliser n’est pas une rêverie. Par contre, penser à cette tâche à d’autres moments, par exemple juste avant de dormir ou durant un long trajet de bus est une rêverie. Chaque affirmation porte sur les rêveries ou sur le fait de rêvasser. Veuillez indiquer pour chacune la réponse qui **vous correspond le mieux en général** en cochant la case appropriée.

1. Je rêvasse: A. Peu fréquemment./B. Une fois par semaine./C. Une fois par jour./D. Quelques fois par jour./E. De nombreuses fois par jour.

2. Les rêveries et le vagabondage de l’esprit représentent: A. 0% de mes pensées de la journée./B. Moins de 10% de mes pensées de la journée./C. Au moins 10% de mes pensées de la journée./D. Au moins 25% de mes pensées de la journée./E. Au moins 50% de mes pensées de la journée.

3. En ce qui concerne les rêveries, je me définirais comme quelqu’un qui: A. Ne rêvasse jamais./B. Rêvasse très rarement./C. Rêvasse occasionnellement./D. Rêvasse modérément./E. Est un rêveur invétéré.

4. Je me rappelle de mes rêveries ou je réfléchis à mes rêveries: A. Peu fréquemment./B. Une fois par semaine./C. Une fois par jour./D. Quelques fois par jour./E. De nombreuses fois par jour.

5. Quand je ne prête pas beaucoup d’attention à un travail, à un livre ou à la tv, j’ai tendance à rêvasser: A. 0% du temps./B. 10% du temps./C. 25% du temps./D. 50% du temps./E. 75% du temps.

6. A la place de faire attention aux gens et aux évènements autours de moi, je passe: A. 0% de mon temps perdu dans mes pensées./B. Moins de 10% de mon temps perdu dans mes pensées./C. Au moins 10% de mon temps perdu dans mes pensées./D. Au moins 25% de mon temps perdu dans mes pensées./E. Au moins 50% de mon temps perdu dans mes pensées.

7. Je rêvasse au travail ou en cours: A. Peu fréquemment./B. Une fois par semaine./C. Une fois par jour./D. Quelques fois par jour./E. De nombreuses fois par jour.

8. Me souvenir du passé, penser au futur, ou imaginer des évènements inhabituels occupe: A. 0% de mes pensées de la journée./B. Moins de 10% de mes pensées de la journée./C. Au moins 10% de mes pensées de la journée./D. Au moins 25% de mes pensées de la journée./E. Au moins 50% de mes pensées de la journée.

9. Je me perds dans des rêveries: A. Peu fréquemment./B. Une fois par semaine./C. Une fois par jour./D. Quelques fois par jour./E. De nombreuses fois par jour.

10. Je rêvasse à chaque fois que j’ai du temps liber: A. Jamais./B. Rarement./C. Parfois./D. Fréquemment./E. Toujours.

11. Je rêvasse au lieu de faire attention lorsque j’assiste à une réunion ou à un spectacle qui n’est pas très intéressant: A. Jamais./B. Rarement./C. Parfois./D. Fréquemment./E. Toujours.

12. Je rêvasse lors d’un long trajet en bus, train, ou avion: A. Jamais./B. Rarement./C. Parfois./D. Fréquemment./E. La plupart du temps

### B Future self thoughts questionnaire

**Table:**

Veuillez indiquer dans quelle mesure chacun des énoncés suivants décrit votre manière de penser ou d’agir, en encerclant un chiffre entre 1 (pas du tout vrai pour moi) et 6 (tout à fait vrai pour moi). Il n’y a pas de bonne ou de mauvaise réponse, veuillez décrire ce qui est vrai pour vous.

1. Mon futur me paraît vague et incertain.

2. Je m’imagine souvent dans le futur de différentes façons et je pense aux diverses voies qui pourraient me conduire à ces différents futurs.

3. Je trouve vraiment difficile de prédire ce à quoi je pourrais ressembler dans le futur.

4. Le fait de penser à moi dans le futur provoque souvent en moi de fortes émotions (qu’elles soient de joie ou de tristesse).

5. Les images que j’ai de moi dans le futur sont très floues, pas claires du tout.

6. Lorsque je rêvasse, je me vois souvent tel que je pourrais être dans le futur.

7. Je passe fréquemment du temps à penser à ce que je pourrais être dans des périodes futures de ma vie.

8. J’ai tendance à m’égarer dans des pensées où j’imagine des futurs possibles pour moi.

9. Lorsque je m’imagine dans le futur, je vois des images claires et vivaces.

10. Mon futur est trop incertain pour que je puisse faire des projets très longtemps à l’avance.

11. J’ai tendance à penser à ce que je pourrais être dans le futur, même quand je ne souhaite pas penser à cela.
